# Parastomal Gallbladder Herniation as an Incidental Preoperative Computed Tomography Finding

**DOI:** 10.1155/2021/8864347

**Published:** 2021-02-11

**Authors:** Magdalini Smarda, Konstantinos Manes, Dimitrios Fagkrezos, Dimitrios Argiropoulos, Konstantinos Laios, Charickleia Triantopoulou, Petros Maniatis

**Affiliations:** ^1^Department of Computed Tomography and Interventional Radiology, “Konstantopouleion” General Hospital of Nea Ionia, Athens, Greece; ^2^Department of Surgery, “Konstantopouleion” General Hospital of Nea Ionia, Athens, Greece; ^3^Department of Radiology, “Konstantopouleion” General Hospital of Nea Ionia, Athens, Greece

## Abstract

A 65-year-old woman with a long surgical history was referred to our hospital's Colorectal Unit for ileostomy management. The patient retained an ileostomy for almost a decade after a series of complicated operations she had undergone, which had several side effects such as electrolyte imbalances, high output, weight loss, and a parastomal hernia. Our hospital's colorectal surgeon proposed to replace the ileostomy with a permanent sigmoidostomy and asked for an imaging evaluation of the parastomal hernia content before the surgery. A computed tomography of the abdomen was performed using our Computed Tomography Department's 64-detector row CT scanner after oral administration of contrast media, without intravenous contrast media injection due to allergy. Concerning the parastomal ileostomal hernia, besides small bowel loops with intraluminal gastrografin, inside the parastomal hernial sac, there also was an almost rounded cystic lesion. Absence of the gallbladder at its typical position and no record of cholecystectomy raised suspicion for gallbladder projection inside the sac. Our suspicion was confirmed during the surgery. Nonexisting acute cholecystitis allowed easy reduction of the gallbladder along with the small bowel loops inside the peritoneal cavity, without proceeding to cholecystectomy at the same time. Finally, ileostomy was annulated and an end colostomy was established. Four days after the surgery, the patient was discharged from the hospital and was happy to live an almost normal life thereafter.

## 1. Introduction

We present the case of a 65-year-old female patient, whose parastomal hernia was complicated with the gallbladder being part of the content within the hernial sac. Unlike most of the reported cases in literature (8 reported worldwide till this moment), our patient did not present to our department with incarceration of the hernia or acute cholecystitis.

## 2. Case Presentation

**Figure 1:**
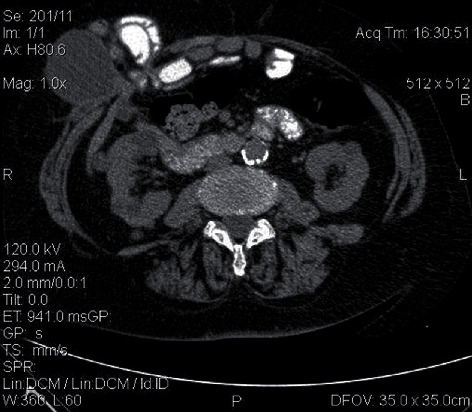
Axial CT slice of the abdomen revealing a cystic lesion within the hernial sac among small bowel loops.

**Figure 2:**
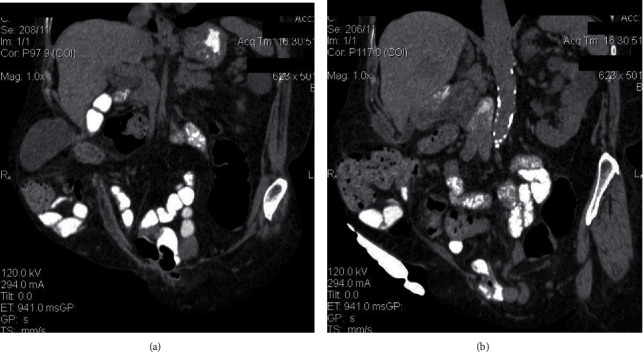
Coronal oblique MPR images showing the parastomal hernial sac content (small bowel loops and a cystic formation). The cyst presenting within the sac in (a) continues with the tubular structure shown in (b), and it represents the gallbladder and the bile duct, respectively. In (b), the ileostomy is also presented.

A 65-year-old woman, who had already been operated several times, was referred to the Colorectal Unit of our hospital for consultation. Her first operation took place ten years ago for surgical management of a rectocele. More specifically, a gynecologist performed a rectopexy to the patient, but unfortunately, the operation was complicated with a rectovaginal fistula. A diverting colostomy at that time was the bridging treatment for a future effort for permanent therapy. This was attempted with a low anterior resection plus a prophylactic loop ileostomy and excision of the fistula. Once more, the postoperative course was not the proper one with rupture of the anastomosis and urgent reoperation in a septic field. Finally, a pull-through procedure was the salvage. The loop ileostomy remained intact, and that exactly was the problem. The patient had a remaining ileostomy for about 9 years, with all the side effects and complications this could have: electrolyte depletion, high output, severe weight loss, and lately, an existing parastomal hernia. So, the patient finally presented to our hospital's Colorectal Department for ileostomy annulation and with her consent, our colorectal surgeon decided to replace it with a permanent sigmoidostomy instead. Before the operation, an imaging evaluation of the parastomal hernia with an abdominal computed tomography (CT) was ordered. The examination was performed using our Computed Tomography Department's 64-detector row CT scanner (Brilliance, Philips Healthcare, Cleveland, OH, USA) with CT slices of 3 mm after oral administration of contrast media. Intravenous iodinated contrast media was not used due to reported allergic reaction of the patient in the past. Coronal, sagittal, and oblique multiplanar reconstructions (MPR images) were also evaluated additionally to axial images. Inside the parastomal hernial sac, apart from small bowel loops, CT revealed a cystic lesion of 8 × 3.5 cm (Figures 1, 2(a) and 2(b)). Nonexistence of the gallbladder at its typical anatomical position and nonreported history of cholecystectomy raised the suspicion of gallbladder projection inside the hernial sac. Intraoperatively, in the context of ileostomy reversal, the hernial sac was identified, dissected, and inspected. Indeed, the gallbladder presented among small bowel loops within the sac. There were no signs of inflammation, leading to an easy reduction of the gallbladder as well as the bowel loops into the peritoneal cavity. Since there was no acute cholecystitis, it was decided not to proceed to a cholecystectomy complicating the operation. The rest of the operation was performed uneventfully, and an end colostomy was established. The parastomal hernia neck was sutured, and no mesh was used for reinforcement of the abdominal wall, as the site was considered potentially infective. The patient recovered well and was discharged from hospital four days after the operation. At her 6-month follow-up, she had incisional hernias at both the midline incision and at the previous ileostomy site. Nevertheless, she was happy to live a normal life at last.

## 3. Discussion

As already mentioned, this is an unusual case of parastomal gallbladder herniation, unexpectedly found during preoperative CT abdominal imaging in a patient coming for surgical management of a remaining ileostomy.

To our knowledge, this is an extremely rare condition since there are only 8 such reported cases around the world up to now [1–8]. In fact, our case is different from the vast majority, because it was not complicated with inflammation or incarceration and therefore constituted an incidental finding during presurgical imaging evaluation.

Noteworthy is also the fact that despite her long surgical history of about a decade and consequently despite lots of previous imaging examinations, our patient's parastomal gallbladder herniation was never mentioned until she came to our hospital.

According to the existing literature, this rare type of parastomal organ projection seems to occur in elderly patients, aging 75-year average [1–8]. Elderly predominance could be attributed to their elastic tissue, reduced peritoneal fat, smaller liver, and increased gallbladder mesentery [9]. Even concerning this observation though, our case is different in the fact that our patient was 10 years younger. Concerning male or female sex predominance, this is the fourth woman reported in a total of 9 patients [1–8]. Therefore, there does not seem to be gender correlation so far, despite the fact that parastomal gallbladder herniation mainly involved elderly men in the first reported cases.

Finally, it should be pointed out that our clinical case has a lot in common with the case presented by Rogers et al. (J Surg Case Rep, 2019), as both patients were women with no signs of acute cholecystitis or incarceration of the herniated gallbladder and so, it did not require urgent intervention with cholecystectomy [8]. Our main difference is that herniation of the gallbladder was incidentally found in our case during preoperative imaging in order to plan an ileostomy reversal rather than a parastomal hernia repair.

To conclude, parastomal gallbladder herniation is an interesting condition because of its rarity, occurring so far in elderly people and usually complicated with cholecystitis or incarceration. Absence of such situations and appearance at a younger age makes our case presentation particularly zestful.
